# Listeria Meningitis in a Patient With Plaque Psoriasis on Ustekinumab Therapy

**DOI:** 10.7759/cureus.23336

**Published:** 2022-03-20

**Authors:** Moni Roy, Naveen Vuppuluri, Ashish K Roy

**Affiliations:** 1 Internal Medicine, University of Illinois College of Medicine, Peoria, USA; 2 Internal Medicine, OSF Saint Francis Medical Center, Peoria, USA; 3 Internal Medicine, Kettering Medical Center, Kettering, USA

**Keywords:** immunocompromised hosts, psoriasis, ustekinumab, meningitis, listeria

## Abstract

Listeria monocytogenes is a foodborne infection and is a leading cause of meningitis. The at-risk population includes patients over age 65, neonates, pregnant females, and patients with impaired cell immunity. Ustekinumab is a human monoclonal antibody that binds to and interferes with the proinflammatory cytokines, interleukin-12 (IL-12) and interleukin-23(IL-23). The drug is used for the treatment of Crohn’s disease, ulcerative colitis, psoriatic arthritis, and plaque psoriasis. We present a case of listeria meningitis in a patient on ustekinumab therapy for plaque psoriasis.

## Introduction

Listeria meningitis is a serious infection with high mortality ranging from 17% to 51% even with appropriate antibiotics [1]. About 0.1-10 cases of listeriosis per million persons are annually reported worldwide [2]. Ustekinumab is a human monoclonal antibody that binds to the p40 subunit shared by IL-12 and IL-23 affecting cell immunity and leading to increased risk of listeria meningitis. Early initiation of antibiotic coverage for Listeria in a patient with risk factors including advanced age, neonates, pregnancy, and on such monoclonal antibody therapy affecting cell-mediated immunity is recommended [3]. We present an interesting case of listeria meningitis in a patient with a suppressed immune system on ustekinumab therapy.

## Case presentation

A 65-year-old male with a past medical history of hypertension and plaque psoriasis on ustekinumab treatment initially presented to the hospital with acute onset nausea, vomiting, abdominal pain for two days. He had been diagnosed with psoriasis five years ago and compliant with 45 mg subcutaneous injections of Ustekinumab every 12 weeks after initial dosing without previous complications. He reported no diarrhea, urinary symptoms, cough, or sore throat. He reported exposure to unwashed produce prior to his presentation. Neck pain and headache were absent on the initial presentation. Severe acute respiratory syndrome coronavirus (SARS-COVID-19) molecular and polymerase chain reaction (PCR) testing returned negative. Complete blood count showed no leukocytosis but had a left shift with 82.3% neutrophils. The basic metabolic profile was normal. He was initially discharged home from the emergency room to return within 24 hours with a right temporal headache and lethargy. He was febrile with a temporal temperature of 102F. No focal neurological deficits were noted on examination. No nuchal rigidity was noted, with Brudzinki’s and Kernigs signs negative on presentation. Urinalysis was normal, chest x-ray showed no acute consolidation. C-reactive protein was elevated at 16 (normal range- <0.5 mg/dL) and ESR was 32 (normal range <20 mm/hr). Computed tomography of the brain did not reveal any acute intracranial abnormalities.

**Figure 1:**
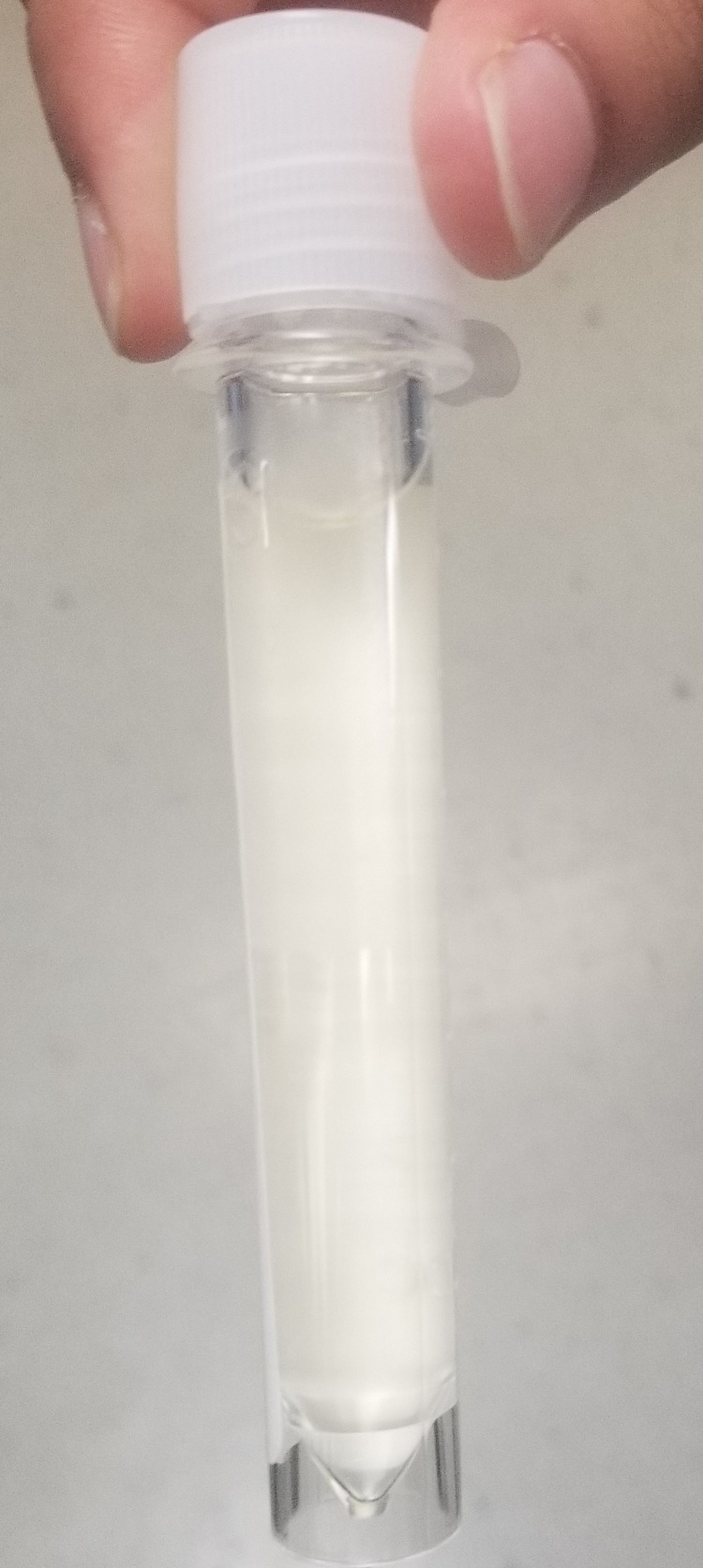
Cloudy appearance of cerebrospinal fluid

Due to increased lethargy, headache, age, and patient being on ustekinumab there was a concern for central nervous system infection. The patient was admitted and started on empirical antibiotic coverage for meningitis with ceftriaxone, vancomycin, ampicillin, and acyclovir. Lumbar puncture was recommended but not done on admission until day 3 due to challenges obtaining consent from the patient. It revealed cloudy cerebrospinal fluid (CSF) (Figure 1).

**Figure 2:**
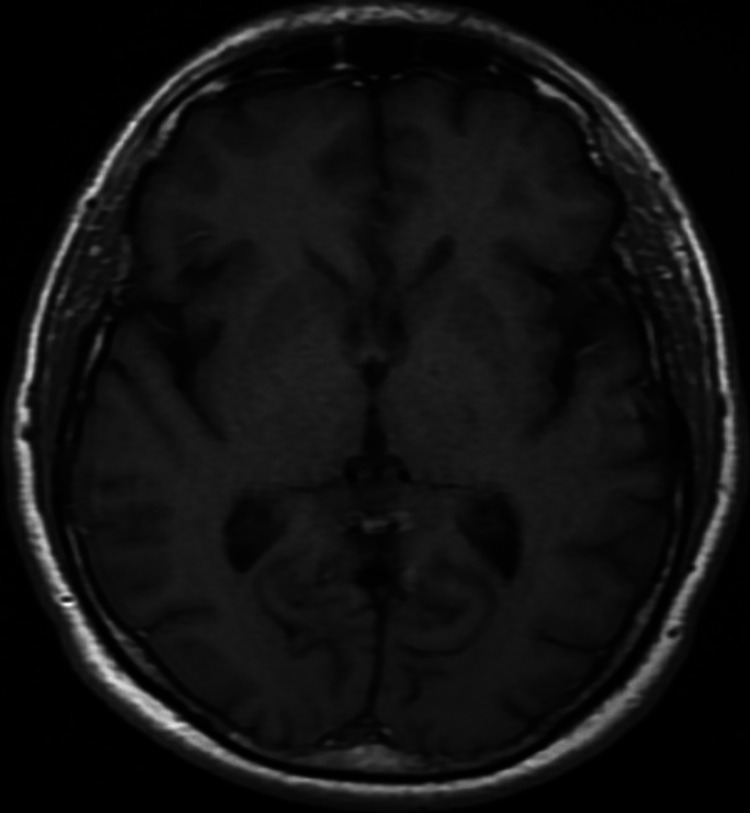
Normal magnetic resonance image of the brain

Blood cultures were obtained before initiation of antimicrobial treatment and one out of two cultures grew gram positive bacilli, later identified as listeria monocytogenes. CSF studies showed 1492/mm(3) nucleated cells, with 48% neutrophils, 47% lymphocytes, 5% monocytes. Glucose was low at 25 mg/dL, and protein elevated at 218 mg/dL. CSF cultures had no growth. Due to inability to obtain a CSF sample prior to initiation of antibiotics, PCR testing was performed on CSF fluid, this returned positive for Listeria monocytogenes. HSV simplex PCR was negative. Antimicrobial treatment was narrowed down to ampicillin and gentamicin. During hospitalization the patient initially had increasing lethargy, and new onset facial droop. Due to concern for brain abscess and possible nerve palsy, magnetic resonance image (MRI) of the brain was obtained which was normal (Figure 2). The patient eventually started improving with gradual resolution of symptoms. He received a total of seven days of gentamicin and was discharged home on ampicillin monotherapy for a total duration of four weeks.

## Discussion

Listeria monocytogenes is primarily a foodborne pathogen and a leading cause of meningitis [4]. It has a tropism for the central nervous system and can cause meningitis, meningoencephalitis, rhombencephalitis, and brain abscesses [5]. The disease carries a high mortality rate of 17%-51% [1]. Nuchal rigidity may be less common as compared to other bacterial meningitis as seen in our patient. CSF studies show pleocytosis, typically neutrophil-predominant but may be mixed with monocytes or lymphocytes and may be difficult to interpret if partially treated with antibiotics or antivirals prior to obtaining sample [6,7]. Ampicillin, 2 g IV every four hours should be added to empiric meningitis coverage in suspected high-risk cases, and once confirmed addition of gentamicin for the first seven to 10 days is recommended as aminoglycosides demonstrated in vitro synergy but the clinical benefit is unclear [8]. No randomized controlled trials exist to direct antibiotic selection [8]. Treatment is recommended for more than 21 days [8]. 

Ustekinumab binds and interrupts messaging of proinflammatory cytokines IL-12 and IL-23 [7]. These cytokines participate in natural killer cell activation, CD4+ T-cell differentiation, and activation. It also interferes with monocyte chemotactic protein-1 (MCP-1), which inhibits communication with and recruitment of additional inflammatory cells. Tumor necrosis factor-alpha (TNF-a), interferon-inducible protein 10 (IP-10), and interleukin 8 (IL-8) are also affected by ustekinumab. Inhibiting the CD4+ T cell activation affects the functionality of the adaptive immune system. This inhibits macrophage recruitment and subsequent antigen-presenting cell (APC) activity and B cell differentiation. The medication subsequently inhibits phagocytic activity and decreases the ability to present particles to antigen-presenting cells in order to form a specific pathogen-directed immune response. Subsequently, adaptive immunity is unable to respond to infection as the APC activity is reduced. As a result of impaired immunity, an infection rate of 27%-72% has been reported in patients taking this medication [9].

Ustekinumab is a monoclonal antibody indicated for moderate to severe inflammatory bowel disease (IBD), plaque psoriasis, and active psoriatic arthritis. In moderate to severe plaque psoriasis dose of 45-90 mg initially followed by doses, every 12 weeks is recommended [9]. The most common reported side effects include diarrhea, vomiting, nausea, and headache. These common side effect symptoms can raise confusion and healthcare providers have to keep a low index of suspicion for suspected systemic infection. It is possible the risk of central nervous infection is dose related and it is within reason to assume that increasing the dose would lead to further immune suppression and decreased innate immunity. This would make patients more prone to develop CNS infections, though with a paucity of cases at this time we cannot support our speculation based on literature. Due to its effect on cell immunity, infections have been reported but based on placebo-controlled clinical trials serious infections have been reported in only 0.3%-2.8% of subjects [9]. Reported serious infections while on ustekinumab for psoriasis include diverticulitis, cellulitis, pneumonia, appendicitis, cholecystitis, sepsis, osteomyelitis, viral infections, gastroenteritis, and urinary tract infections [9]. There has been one reported case of listeria meningitis in a patient with Crohn's disease treated with ustekinumab [9]. Of note, the dose requirement for induction in Crohn's disease is 260-520 mg followed by a maintenance dose every eight weeks [10,11]. Re-initiation of immunosuppressants in patients who have had complications of infection is a difficult decision and shared decision making between the physician and patient is required after discussion of risks and benefits.

## Conclusions

As per our literature review, this is the first reported case of listeria meningitis in a patient with plaque psoriasis on ustekinumab treatment. In our patient exposure to unwashed produce prior to presentation, most likely led to listeria exposure, and the presence of risk factors including age and compromised cell-mediated immunity led to a serious infection.
